# Liver resection versus radiofrequency ablation for hepatocellular carcinoma: A systemic review and meta-analysis

**DOI:** 10.3389/fonc.2025.1607338

**Published:** 2025-09-09

**Authors:** Zheng He, Guolang Song, Guangchao Yang, Xuan Fu, Meng Tian, Yanhui Zhu

**Affiliations:** Department of General Surgery, Shenzhen Baoan Shiyan People’s Hospital, Shenzhen, Guangdong, China

**Keywords:** meta-analysis, hepatectomy, radio frequency ablation, hepatocellular carcinoma, liver cancer

## Abstract

**Background:**

Liver resection and radiofrequency ablation (RFA) are two common treatments for hepatocellular carcinoma (HCC). However, their efficacy and safety remain unclear. We aimed to conduct a systematic review and meta-analysis to compare the effectiveness and safety of these two treatments.

**Methods:**

We searched multiple databases to identify randomized controlled trials (RCTs) that compared liver resection with RFA for the treatment of HCC. The primary outcome was 5-year overall survival rate. The secondary endpoint was the incidence of complications. We used RevMan 5.4 software to calculate the pooled effects and 95% confidence interval (CI).

**Results:**

Ten RCTs and 35 cohort studies were included in this meta-analysis. The pooled OR for 5-year overall survival rate favored liver resection (OR = 1.76, 95% CI = 1.19-2.61, *P*<0.00001). RFA was indicated with less postoperative complications (OR = 3.35, 95% CI = 2.52-4.45, *P*<0.00001).

**Conclusion:**

This meta-analysis suggests that liver resection is more effective than RFA in treating HCC with regard to higher 5-year overall survival rate, while the safety of liver resection was concerning. We recommend liver resection as a first-line treatment for HCC, but RFA may be a preferable choice for patients who are not suitable for surgical procedures. More high-quality RCTs are needed to confirm these findings.

**Systematic review registration:**

https://www.crd.york.ac.uk/prospero/, identifier CRD42025458621.

## 1 Introduction

Hepatocellular carcinoma (HCC) is among the most common cancers worldwide, and is associated with high morbidity and mortality rates (1). The primary treatment options for HCC include surgical liver resection (LR) and radiofrequency ablation (RFA) (2). LR involves removing the tumor and surrounding liver tissue; however, compared to RFA, LR may be associated with higher perioperative risks, including morbidity and mortality (3). RFA is a minimally invasive technique that destroys cancer cells using high-frequency alternating currents. It is often used as an alternative to surgical LR, especially in patients with small tumors or contraindications to surgery (4).

LR and RFA are considered to be effective treatments for early stage HCC (5). Recent studies have compared effectiveness and outcomes of LR versus RFA in the treatment of HCC, although with varying results. Some studies have reported that LR results in better survival rates, whereas others have described comparable outcomes between the 2 approaches (6).

Despite various studies comparing the effectiveness of LR and RFA, the findings have not consistently favored one treatment over the other. Consequently, systematic reviews and meta-analyses are needed to provide more robust evidence-based recommendations for the optimal management of HCC (7).

However, there are some limitations to previous meta-analyses, including differences in patient selection criteria, surgical techniques, and outcome measures, which may have affected the results (8). As such, this systematic review and meta-analysis aimed to provide a comprehensive evaluation of the available evidence regarding the effectiveness of LR versus RFA in the treatment of HCC and to address existing limitations in the literature.

## 2 Materials and methods

### 2.1 Literature search

This systematic review and meta-analysis used PubMed database search strategies in accordance with recommendations from the Cochrane Handbook for Systematic Reviews of Interventions, and complied with the Preferred Reporting Items for Systematic Reviews and Meta-Analyses (i.e., “PRISMA”), and Assessing the Methodological Quality of Systematic Reviews (i.e., “AMSTAR) guidelines (9–11). Randomized control trials (RCTs) and cohort studies published before Sep 1, 2024, were included. The search terms were liver resection AND radiofrequency ablation AND hepatocellular carcinoma. The reference lists of all retrieved studies were reviewed for additional, potentially eligible studies. Two authors independently reviewed the titles, abstracts, and full texts according to the inclusion and exclusion criteria, while a third author adjudicated any disagreements.

### 2.2 Study selection and data extraction

Eligible studies compared survival outcomes between LR and RFA. Studies were excluded if overall survival (OS) was not reported. Studies involving overlapping populations have been conducted. Statistically unreliable estimates were avoided by excluding studies with < 10 patients per group. Two researchers independently extracted relevant information using a predefined data extraction sheet. Consensus was reached in discussions to resolve discrepancies and missing data. The mean and standard deviation were estimated using the median and interquartile range (IQR) or median and range (12, 13).

### 2.3 Outcomes

The primary outcome was OS (1-, 3-, and 5-year survival rates). The secondary outcomes were operative duration, postoperative mortality, estimated blood loss (EBL), length of hospital stay, postoperative complications, and recurrence rates.

### 2.4 Risk of bias

All RCTs were critically appraised according to the revised Risk of Bias tool (ROB2.0), and non-randomized studies were evaluated using the ROBINS-I tool (14, 15). The risk of bias was independently assessed by 2 authors and adjudicated by a third when required.

### 2.5 Data analysis

This meta-analysis was performed in accordance with the Cochrane Guidelines for Systematic Reviews (9). A Mantel–Haenszel model was used to calculate odds ratio (OR) and corresponding 95% confidence interval (CI) for categorical data. Continuous data were analyzed using the inverse variance model and expressed as mean difference (MD) with 95% CI. Heterogeneity was assessed using the I^2^ test. A fixed-effects model was used to pool effects. Review Manager version 5.4 and R (R Foundation for Statistical Computing, Vienna, Austria) were used to perform statistical analyses. A *P* value < 0.05 was defined as the threshold for statistical significance of the estimates. This study was registered with The International Prospective Register of Systematic Reviews (i.e., “PROSPERO”) (CRD CRD42025458621).

## 3 Results

**Figure 1:**
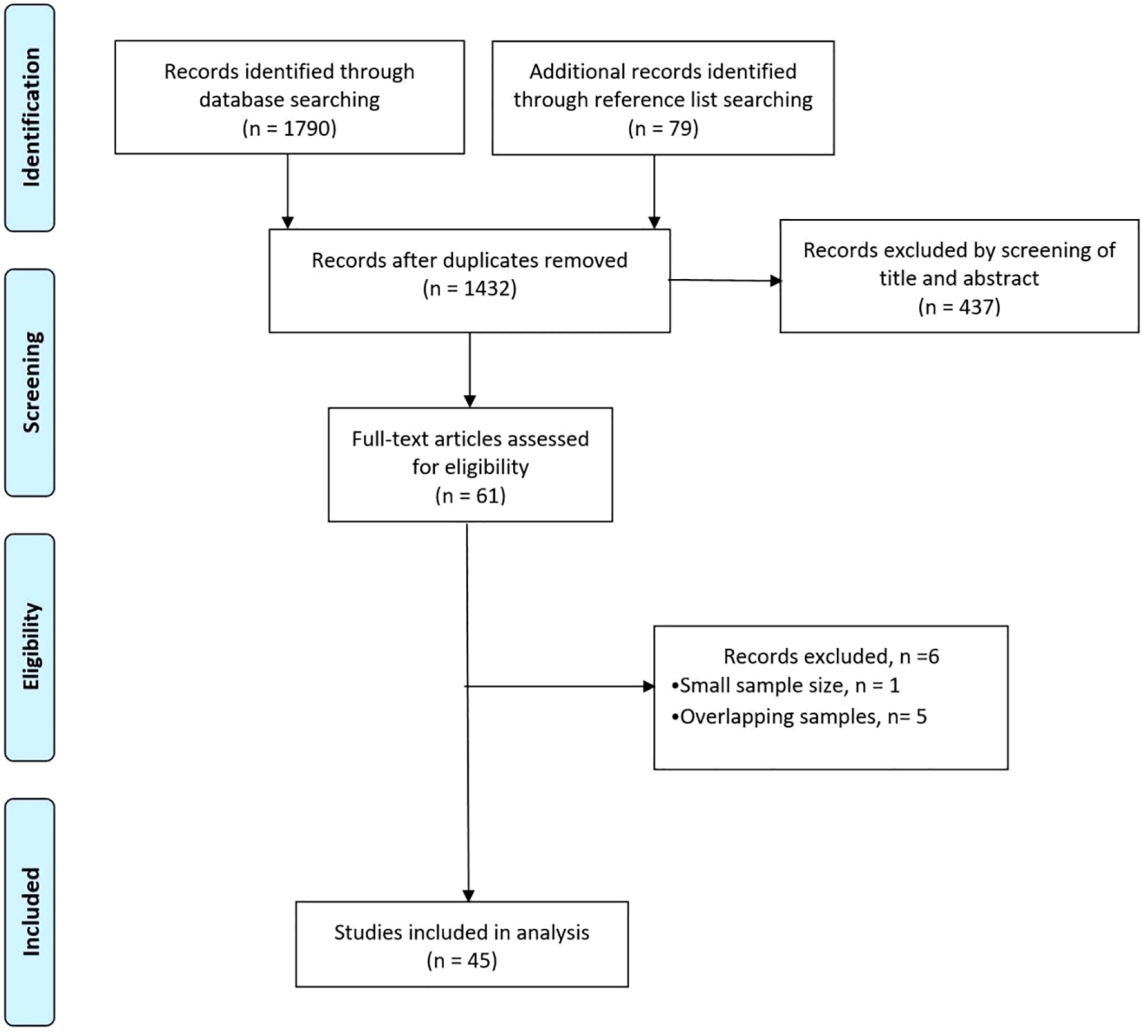
PRISMA flowchart.

The literature search retrieved 1790 studies. After duplicates were removed and titles and abstracts were screened, 1432 studies remained, of which 61 full-text articles were read. In total, 45 studies (14,849 patients; 7567 RFA and 7282 LR procedures) were included in the analysis (**Figure 1**).

A summary of the 45 included studies, of which OS was reported in 39, is presented in **Table 1**. The systematic review included 10 RCTs (16–25) and 35 cohort studies (26–60), with 14 cohort studies using propensity score matching (PSM). Two RCTs demonstrated a high risk of bias, 7 studies indicated some concerns regarding the risk of bias, and 1 study had a low risk of bias. Eight non-randomized studies had a serious risk of bias, and 27 studies had a moderate risk of bias (**Supplementary Figure S1**).

**Table 1:** The basic characteristics of the included studies.

Author	Year	Country	Research type	Number of participants		Age		Follow-up (month)	Outcome measures	Risk of bias
				RFA	SR	RFA	SR			
Chen MS (25)	2005	China	RCT	47	65	52.4	49.2	36	OS; DFS; C	High
Chen MS (24)	2006	China	RCT	71	90	51.9	49.4	29.2	OS; DFS; LOS; M; C	Some concerns
Lu MD (18)	2006	China	RCT	51	54	55	49	40	OS; DFS; LOS; M; C	Some concerns
Huang J (21)	2010	China	RCT	115	115	56.6	55.9	46.4	LOS; M; AE; OS; RFS; TR	Some concerns
Feng K (22)	2012	China	RCT	84	84	51	47	36	OT; EBL; LOS; OS; RFS; TR	Some concerns
Fang Y (23)	2014	China	RCT	60	60	51.4	53.5	40	LOS; C; M; DFS; OS	High
Liu H (19)	2016	China	RCT	100	100	52	49	56	RFS; OS	Some concerns
Ng, KKC (17)	2017	China	RCT	109	109	57	55	93	OT; EBL; M; C; LOS; OS; TR	Some concerns
Lee HW (20)	2018	Korea	RCT	34	29	56.1	55.6	64	OS; DFS; TR	Some concerns
Takayama T (16)	2022	Japan	RCT	151	150	69	68	72	OT; EBL; LOS; RFS; TR;	Low
Zhou Z (40)	2014	China	Cohort	31	21	46.7	42.2	60	C; EBL; LOS; OS; OT	Serious
Lai C (55)	2016	China	Cohort	33	28	62.8	56.5	36	OT; EBL; LOS; TR	Serious
Liu PH (6)	2016	China	Cohort	79	79	64	60	96	OS; TR	Serious
Song J (49)	2016	China	Cohort	78	78	49	49.3	96	OT; EBL; LOS; TR; OS	Moderate
Vitali GC (46)	2016	Switzerland	Cohort	60	45	66.2	59.5	144	C; OT; M; LOS	Moderate
Di Sandro S (59)	2019	Italy	Cohort	91	91	65.5	66	60	OS, TR	Moderate
Cha DI (60)	2020	Korea	Cohort	178	145	56.8	53.3	97.2	C, OS	Serious
Hsiao CY (57)	2020	China	Cohort	231	156	62.2	58.8	84	OS; TR	Serious
Lin CH (52)	2020	China	Cohort	39	36	NA	NA	60	LOS; OT; OS; DFS	Moderate
Tsukamoto M (47)	2020	Japan	Cohort	94	77	67.4	65.2	32.8	OS	Moderate
Wei C (45)	2020	China	Cohort	183	68	70	64	45.1	OS; C	Serious
Yan J (41)	2020	China	Cohort	42	84	48.5	49.4	39.3	OS; DFS	Moderate
Hur MH (56)	2021	Korea	Cohort	194	567	58.3	55.2	81	OS; RFS;	Moderate
Lee D (54)	2021	Korea	Cohort	315	251	60.8	57.5	30	OS; RFS; C;	Moderate
Ogiso S (50)	2021	Japan	Cohort	136	85	73	69	66	LOS; M; C; OS; DSS	Moderate
Wu C (44)	2021	China	Cohort	73	83	NA	NA	84	OS; RFS;	Moderate
Xu H (42)	2021	China	Cohort	46	48	56.3	57.2	24	OT; EBL; Cost; C; OS; RFS	Serious
Lee J (53)	2022	South Korea	Cohort	159	232	NA	NA	64.8	OS; RFS; LOS; AE	Serious
Terashima T (48)	2022	Japan	Cohort	863	863	72	72	36	OS; TR	Moderate
Xie W (43)	2022	China	Cohort	21	46	59.9	54.8	60	OS; RFS	Moderate
Kang TW (35)	2015	Korea	PSM	438	142	56.5	52	96	OS; C; LOS	Moderate
Kim GA (34)	2015	Korea	PSM	331	273	55.4	55.4	72	DDS; RFS; TR	Moderate
Chong CCN (38)	2020	China	PSM	155	59	62.1	57.7	47.2	OS; DFS; C	Moderate
Oh JH (30)	2020	China	PSM	87	48	59	54.5	62.4	OS; RFS; C	Moderate
Pan Y (29)	2020	China	PSM	314	163	57	51	26.2	OS; RFS; C; M; LOS; Cost	Moderate
Conticchio M (37)	2021	Italy	PSM	165	429	75	74.9	60	OS; DFS; C; LOS; OT; M	Moderate
Li Y (31)	2021	China	PSM	85	103	62	57	56	OS; DFS; M; TR	Moderate
Cheng K (39)	2022	China	PSM	69	99	65.5	63.6	34	C; M; LOS; OS; DFS; TR; RFS	Moderate
Delvecchio A (36)	2022	Italy	PSM	40	37	74.5	75	60	OT; C; LOS; M; TR; OS; DFS	Moderate
Kim S (33)	2022	Korea	PSM	264	101	66.5	57.8	57	OS; RFS(DFS); LOS; C; TR	Moderate
KO SE (32)	2022	Korea	PSM	60	29	60	55.8	50	OS; RFS;	Moderate
Zhang C (28)	2022	China	PSM	95	156	58.3	54	96	OS; RFS; OT; LOS	Moderate
Meng F (27)	2021	China	SEER; PSM	524	472	62.8	62.8	144	OS	Moderate
Xie Q (26)	2022	China	SEER; PSM	811	794	NA	NA	60	OS; DSS	Moderate
Eilard MS (58)	2021	Sweden	SweLiv-registry	361	438	NA	NA	65.4	OS; M	Moderate

AE, adverse event; C, complications; DSS, disease specific survival; EBL, estimated blood loss; SR, surgical resection; LOS, length of stay; M, mortality; NA, not available; NOS, Newcastle-Ottawa Scale; OS, overall survival; OT, operative time; PSM, propensity score matching; RFA, radiofrequency ablation; RFS, recurrence free survival; TR, tumor recurrence.

**Figure 2:**
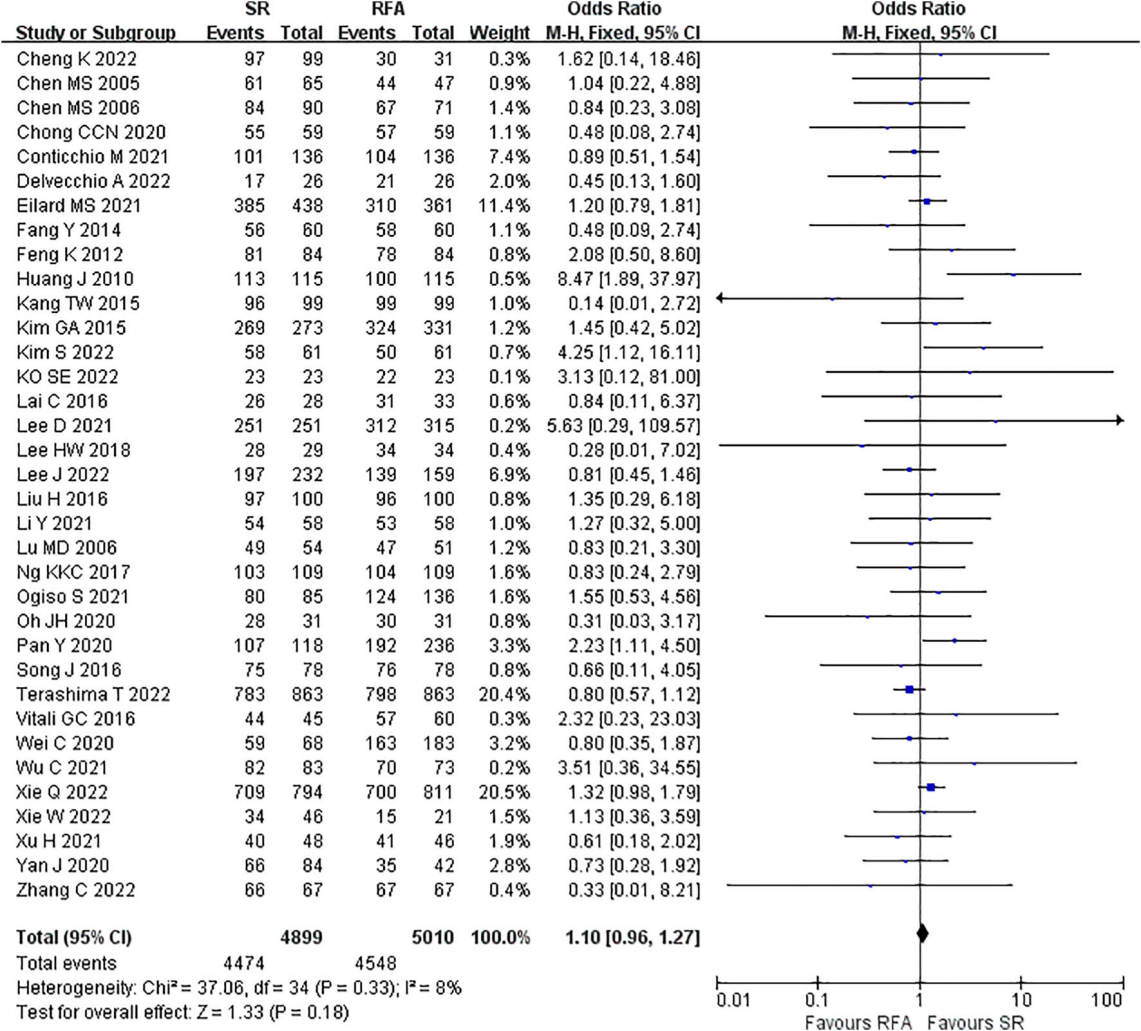
Meta-analysis of 1-year overall survival rate comparing surgical resection with radio frequency ablation.

**Figure 3:**
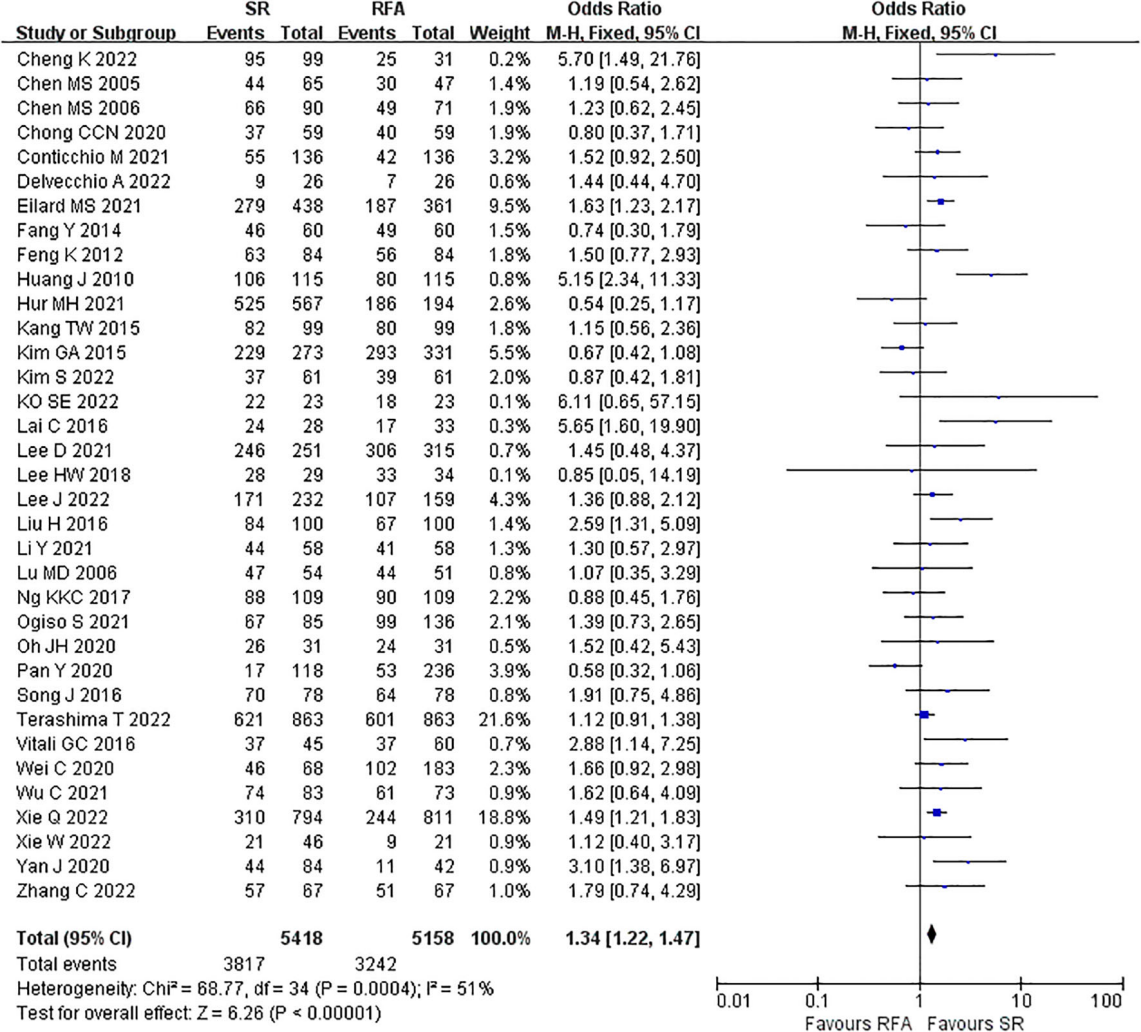
Meta-analysis of 3-year overall survival rate comparing surgical resection with radio frequency ablation.

**Figure 4:**
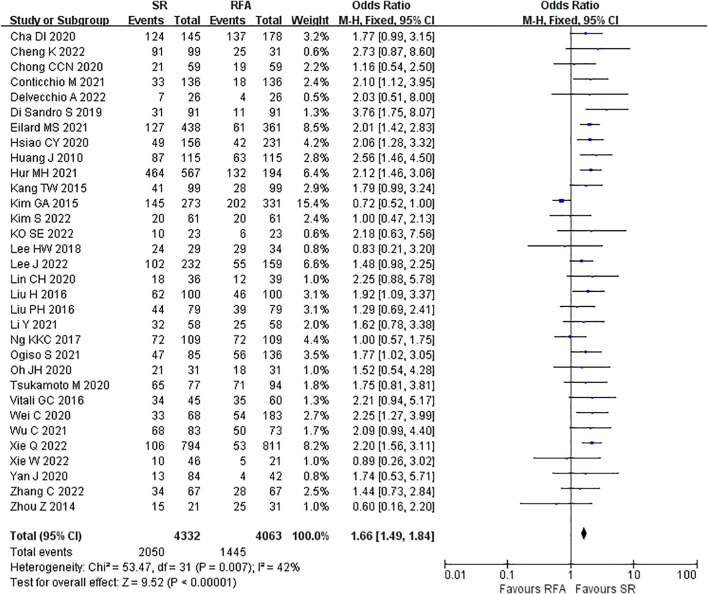
Meta-analysis of 5-year overall survival rate comparing surgical resection with radio frequency ablation.

**Figure 5:**
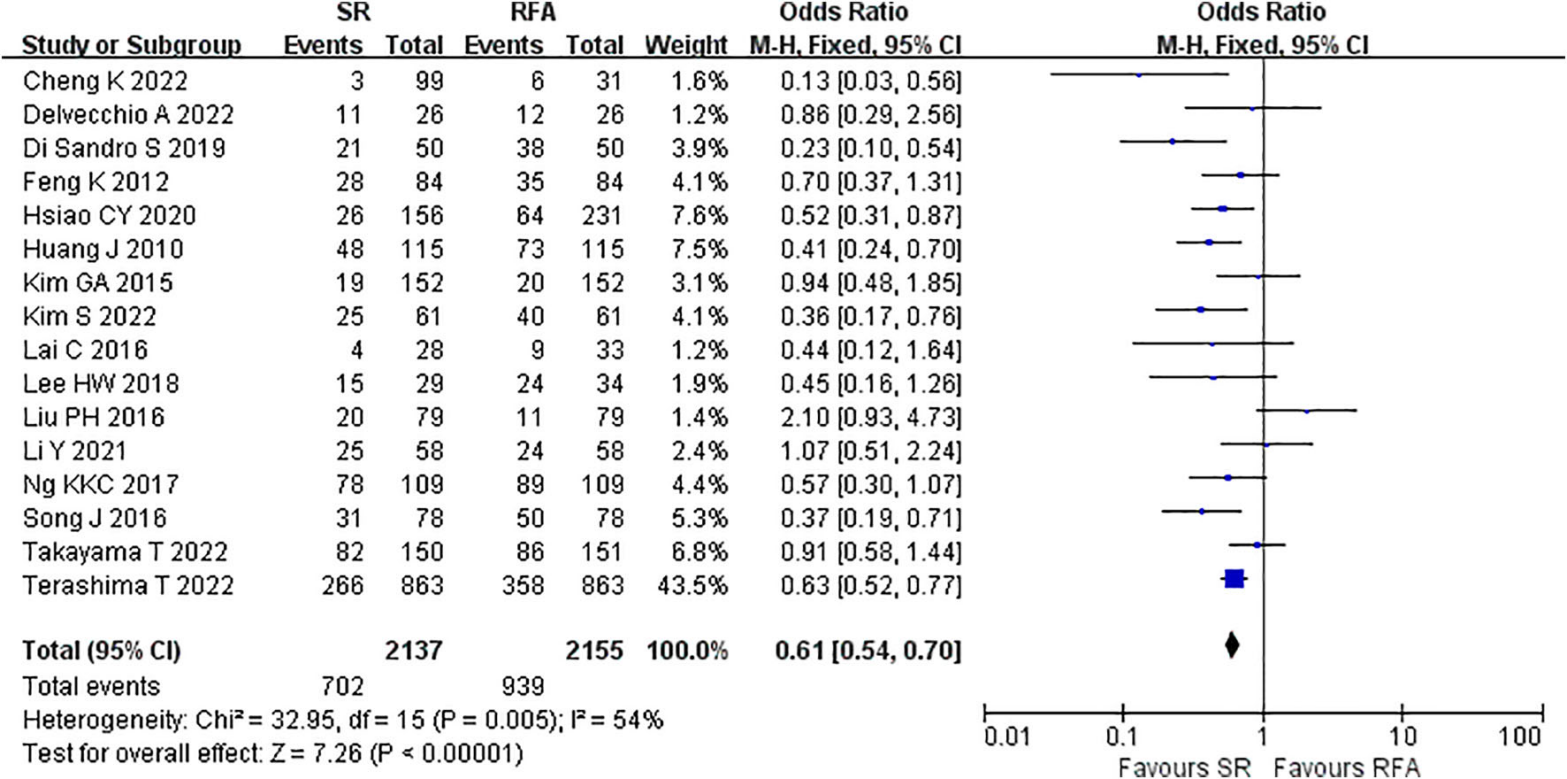
Meta-analysis of recurrence rate comparing surgical resection with radio frequency ablation.

LR significantly prolonged patient survival compared with RFA. The 1-, 3-, and 5-year OS rates were used to compare survival outcomes between RFA and LR. The 1-year OS for RFA and LR was similar (LR versus [*vs*.] RFA, OR 1.10 [95% CI 0.96 – 1.27]; *P* = 0.18, I^2^ = 8%) (**Figure 2**), while LR was associated with better 3-year OS (LR *vs*. RFA, OR 1.34 [95% CI 1.22 – 1.47]; *P*<0.00001, I^2^ = 51%) (**Figure 3**), and 5-year OS (LR *vs*. RFA, OR 1.66 [95% CI 1.49 – 1.84]; *P*<0.00001, I^2^ = 42%) (**Figure 4**) compared with RFA. The recurrence rate for LR was consistently much lower than that of RFA (OR 0.61 [95% CI 0.54 – 0.70]; *P*<0.00001, I^2^ = 54%) (**Figure 5**).

**Figure 6:**
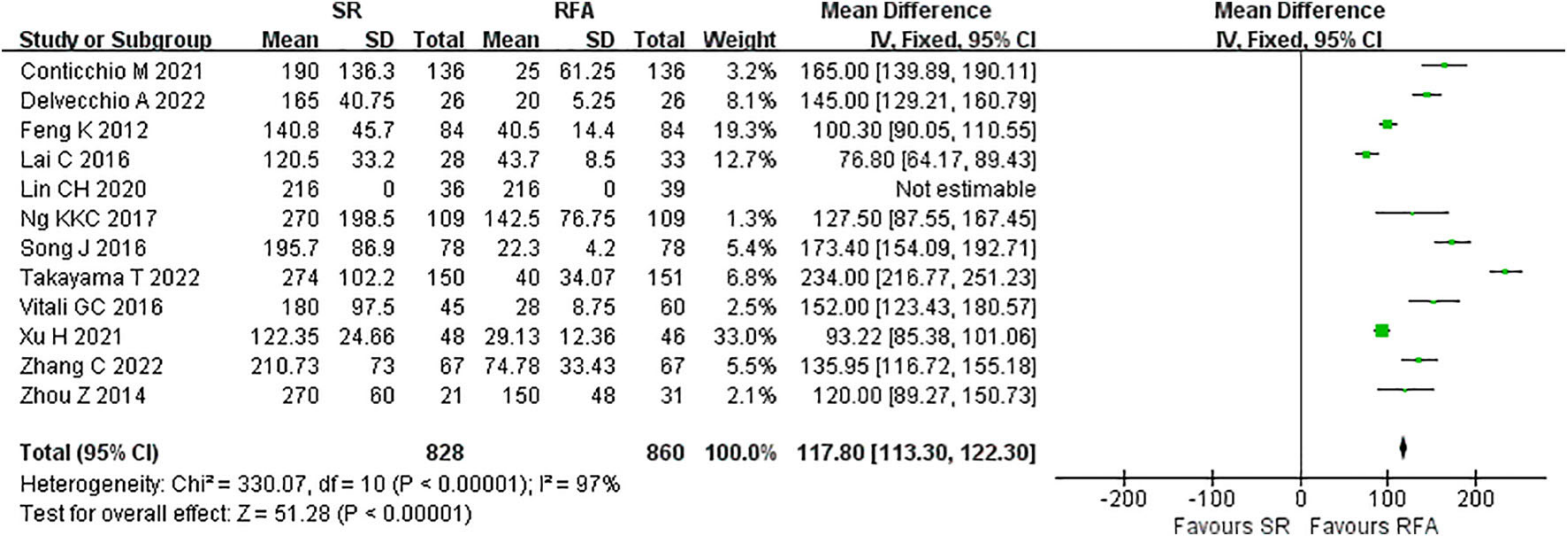
Meta-analysis of operative time comparing surgical resection with radio frequency ablation.

**Figure 7:**
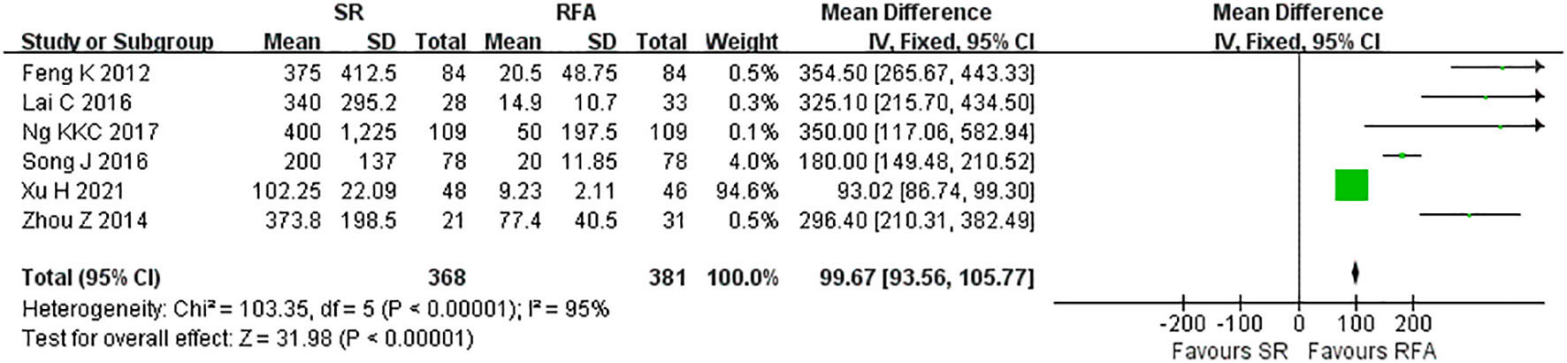
Meta-analysis of estimated blood loss comparing surgical resection with radio frequency ablation.

RFA demonstrated a significant advantage over LR in terms of intraoperative outcomes. Operative duration was significantly shorter in the RFA *vs*. LR groups (LR *vs*. RFA, MD 117.80 [95% CI 113.30 – 122.30]; *P*<0.00001, I^2^ = 97%) (**Figure 6**). EBL was significantly lower in the RFA group than that in the LR group (LR *vs*. RFA, MD 99.67 [95% CI 93.56 – 105.77]; *P*<0.00001, I^2^ = 95%) (**Figure 7**).

**Figure 8:**
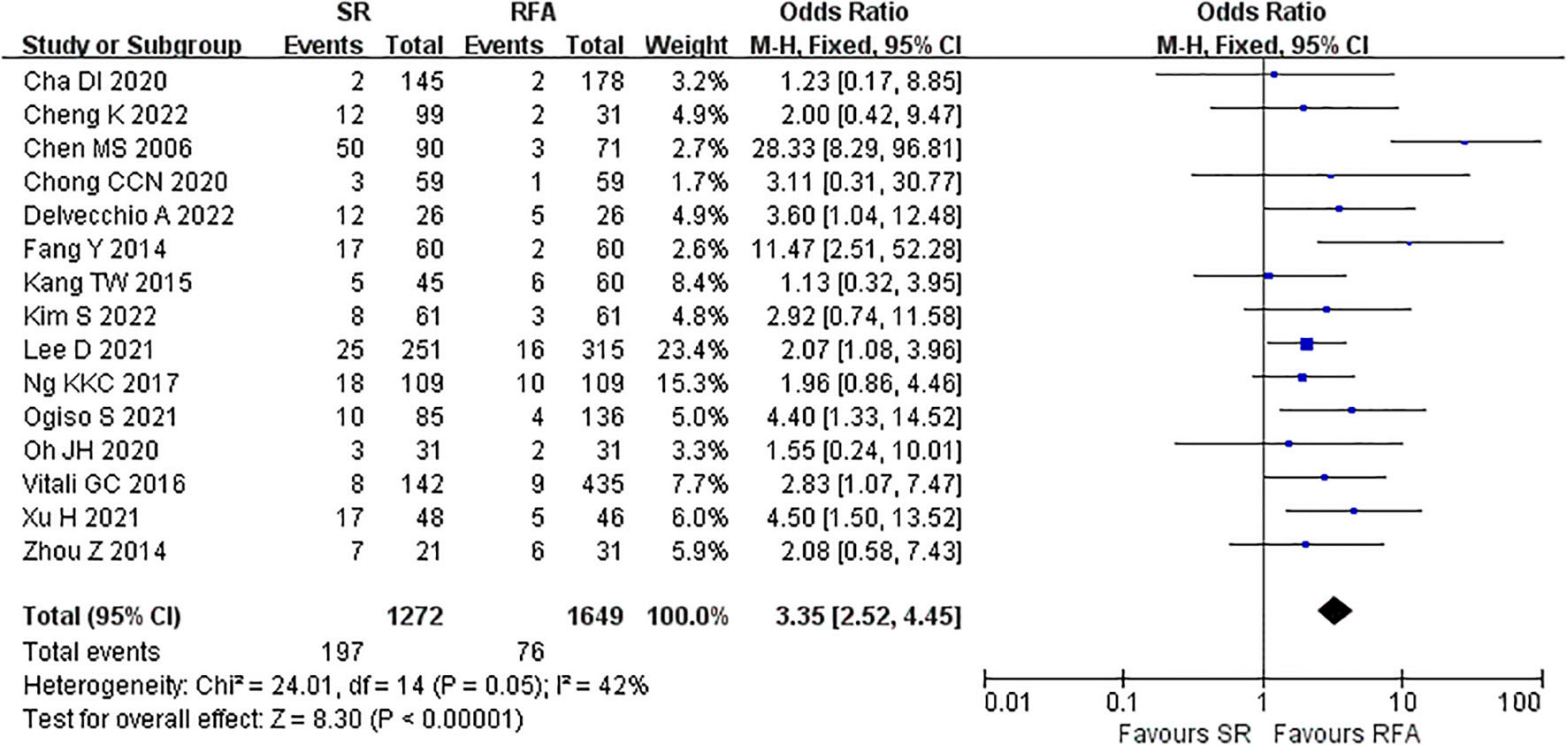
Meta-analysis of postoperative complications comparing surgical resection with radio frequency ablation.

**Figure 9:**
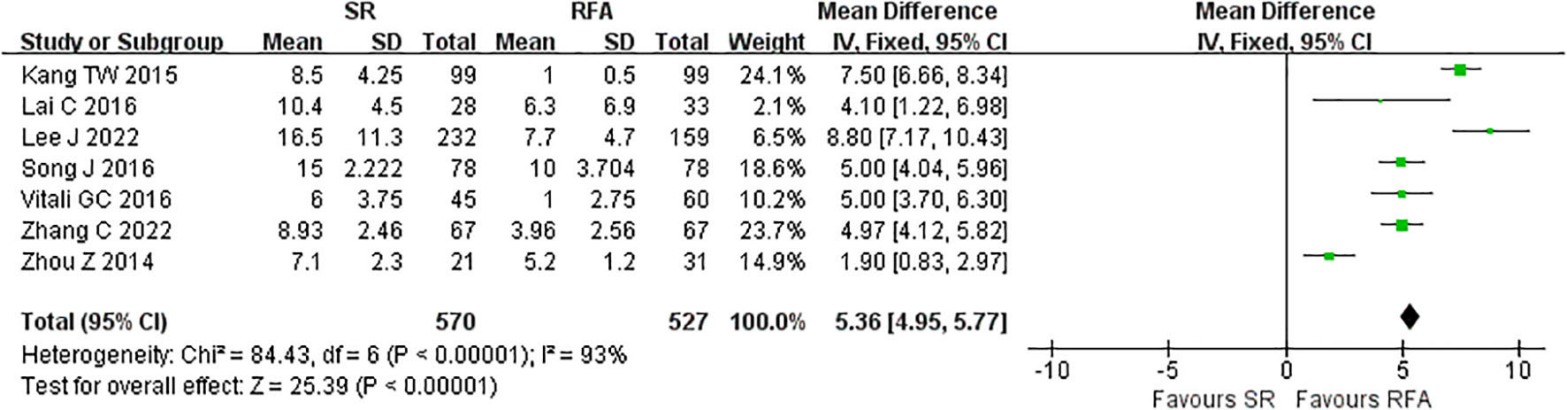
Meta-analysis of postoperative length of hospital stay comparing surgical resection with radio frequency ablation.

**Figure 10:**
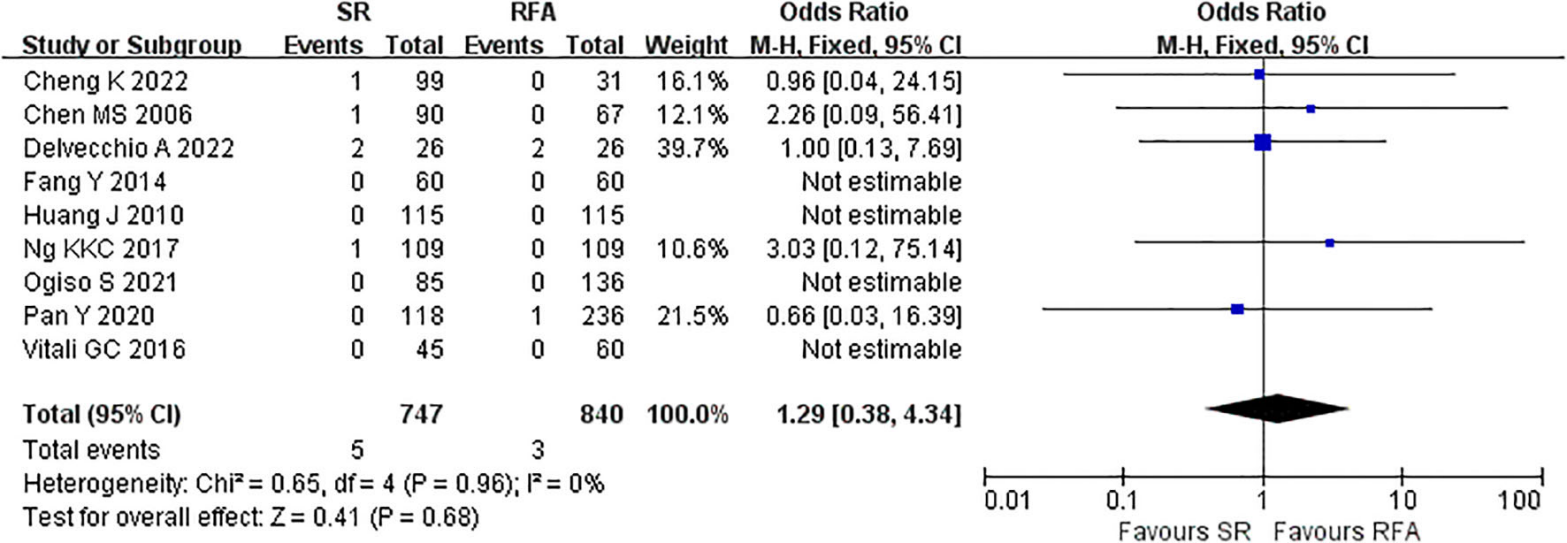
Meta-analysis of mortality comparing surgical resection with radio frequency ablation.

The short-term outcomes of RFA were better than those of LR. The RFA group experienced fewer postoperative complications than the LR group (LR *vs*. RFA, OR 3.35 [95% CI 2.52 – 4.45]; *P*<0.00001, I^2^ = 42%) (**Figure 8**). The postoperative length of hospital stay was consistently shorter in the RFA group (LR *vs*. RFA, MD 5.36 [95% CI 4.95 – 5.77]; *P*<0.00001, I^2^ = 93%) (**Figure 9**). However, mortality rates were similar between the LR and RFA groups (LR *vs*. RFA, OR 1.29 [95% CI 0.38 – 4.34]; *P* = 0.68, I^2^ = 0%) (**Figure 10**).

**Figure 11:**
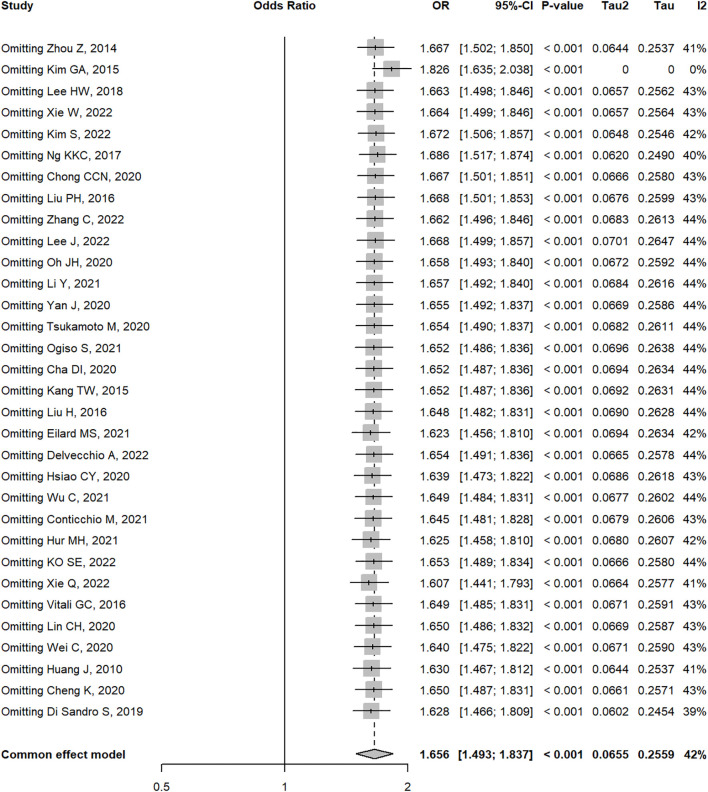
Sensitivity analysis of 5-year overall survival by omitting single studies.

**Figure 12:**
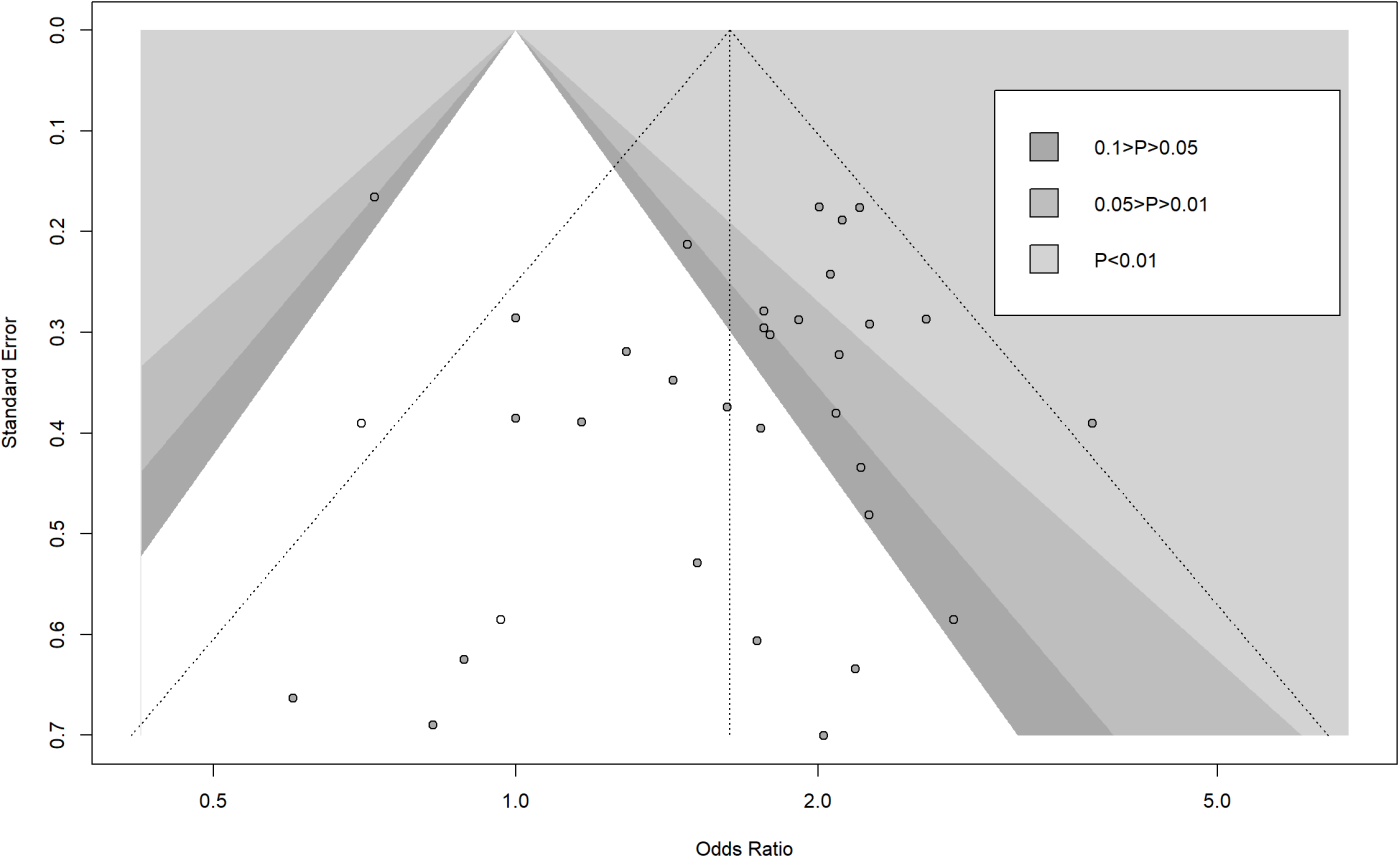
Contour-enhanced funnel plot with trim-and-fill method (white dot) for publication bias of 5-year overall survival.

When the study by Kim (34) was excluded, the OR and 95% CI changed significantly from 1.66 (1.49 – 1.84) to 1.83 (1.64 – 2.04), indicating that the study by Kim (34) was the main source of bias (**Figure 11**). To assess the robustness of primary outcomes, we performed comprehensive sensitivity analyses. Exclusion of studies with high risk of bias and non-propensity-score-matched cohorts consistently demonstrated superior outcomes for liver resection over radiofrequency ablation (OR 1.68, 95% CI 1.20 – 1.94; P<0.01, I²=57.7%, **Supplementary Figure S2**). Similarly, stratification by study design revealed concordant results: analysis restricted to randomized trials maintained significant advantage for resection (OR 1.60, 95% CI 1.08 – 2.37; P<0.0001, I²=40.2%), while observational studies alone yielded comparable effect sizes (OR 1.70, 95% CI 1.44 – 2.00; P<0.0001, I²=44.4%, **Supplementary Figure S3**). These methodologically distinct approaches collectively demonstrate the stability of our core findings across analytical frameworks. Trim-and-fill analysis indicated potential publication bias for the outcome of 5 year overall survival (OS), with imputation of 2 hypothetical studies reducing the HR magnitude from (LR *vs*. RFA, OR 1.66 [95% CI 1.49 – 1.84]; P<0.00001, I^2^ = 42%) to (LR *vs*. RFA, OR 1.63 [95% CI 1.41 – 1.90]; P<0.0001, I^2^ = 44%). While this suggests our pooled effect may overestimate LR’s benefit, the adjusted HR remained statistically significant and clinically relevant. Nevertheless, the possibility of unpublished null findings warrants caution in interpreting the magnitude of survival advantage (**Figure 12**). Moreover, publication bias resulted in asymmetry of the funnel plot.

## 4 Discussion

This systematic review and meta-analysis compared the efficacy and safety of LR and RFA for the treatment of HCC (**Table 2**). Our analysis included 45 studies comprising 14,849 patients, of whom 7567 underwent RFA and 7282 underwent LR. Results of analysis revealed that LR significantly prolonged OS of patients with HCC compared with RFA. The recurrence rate after LR was significantly lower than RFA. The intraoperative outcomes favored RFA, with a significantly shorter operative duration, reduced EBL, fewer postoperative complications, and shorter postoperative length of hospital stay.

**Table 2:** Summary of the pooled effects.

Outcomes	Num. of studies	Num. of patients		Findings (95%CI)	*P* values	*I²*, %
		SR	RFA			
1-year overall survival	35	4899	5010	OR, 1.10 (0.96, 1.27)	0.18	8
3-year overall survival	35	5418	5158	OR, 1.34 (1.22, 1.47)	<0.00001	51
5-year overall survival	32	4332	4063	OR, 1.66 (1.49, 1.84)	<0.00001	42
Recurrence	16	2137	2155	OR, 0.61 (0.54, 0.70)	<0.00001	54
Operative time	12	828	860	MD, 117.80 (113.30, 122.30)	<0.00001	97
Estimated blood loss	6	368	381	MD, 99.67 (93.56, 105.77)	<0.00001	95
Postoperative complications	15	1272	1649	OR, 3.35 (2.52, 4.45)	<0.00001	42
Length of hospital stay	7	570	527	MD, 5.36 (4.95, 5.77)	<0.00001	93
Mortality	9	747	840	OR, 1.29 (0.38, 4.34)	0.68	0

Our meta-analysis revealed that LR was associated with a better OS rate than RFA (61). This finding is consistent with those of several previous investigations. One possible explanation is that surgical LR offers complete tumor removal with sufficient margins to reduce the risk for recurrence (62, 63). However, RFA relies on thermal energy to destroy tumors, which may not be completely effective in eliminating HCC (64). Our results are important for clinical decision-making because they provide support for recommending LR for patients with HCC who are physically able to tolerate invasive surgical procedures.

However, RFA had a superior effect on intra- and postoperative outcomes compared with LR. Our study and several RCTs suggest that RFA minimizes operative duration and reduces intraoperative EBL (65). This finding may have important implications, especially in reducing operative risk in patients with poor liver function, performing repeated treatments, or managing more challenging lesions, such as large tumors or those located near vital structures (66). In addition, our findings demonstrated that RFA resulted in shorter hospital stays and fewer postoperative complications. These are important benefits for improving patient outcomes and reducing healthcare costs (67).

One of the strengths of our study is its large sample size, which provides robust data for the comparison between LR and RFA in the treatment of HCC. We also included high-quality studies that minimized the impact of bias and increased the reliability of the results (16). Furthermore, although the positive results from the sensitivity and publication bias analyses suggested that there may have been some degree of bias, the fact that the conclusion of the meta-analysis remained favorable for long-term survival after bias adjustment indicates that the conclusion of this meta-analysis is robust.

However, this study also had several limitations. First, the heterogeneity of the included studies may have affected the consistency of findings. Second, although we performed a subgroup analysis to reduce heterogeneity, results may have been affected due to the various surgical techniques and devices used. Although our findings demonstrate LR’s survival advantage in broad HCC populations, further research is needed to clarify its benefit in specific clinical scenarios—particularly among elderly patients, those with marginal liver reserve (Child-Pugh B), or complex tumor locations where RFA’s minimally invasive profile may offset oncologic trade-offs. Future individual patient data meta-analyses or propensity-matched cohort studies targeting these subgroups are warranted.

## 5 Conclusions

In conclusion, based on pooled evidence from randomized and high-quality observational studies, liver resection demonstrates superior survival outcomes compared to RFA, particularly for patients with preserved liver function and resectable tumors. However, given the inherent selection bias in non-randomized comparisons and heterogeneity in patient populations, treatment decisions should be individualized, considering comorbidities, tumor location, and local expertise. LR represents a preferred curative-intent option where clinically feasible, rather than a universal ‘first-line’ approach.

## Data Availability

The original contributions presented in the study are included in the article/**Supplementary Material**. Further inquiries can be directed to the corresponding author.

## Supplementary material

Supplementary Figure 1 Risk of bias evaluation.

Supplementary Figure 2 Sensitivity analysis of primary outcomes after exclusion of high-risk-of-bias studies and non-propensity-score-matched cohorts.

Supplementary Figure 3 Stratified analysis by study design showing maintained advantage of liver resection in both randomized trials and observational studies.
