# Supplementary figures and images for: Liver resection versus radiofrequency ablation for hepatocellular carcinoma: A systemic review and meta-analysis
Source: Front Oncol. 2025 Sep 9;15:1607338. doi: 10.3389/fonc.2025.1607338 (PMC12455236; doi:10.3389/fonc.2025.1607338)

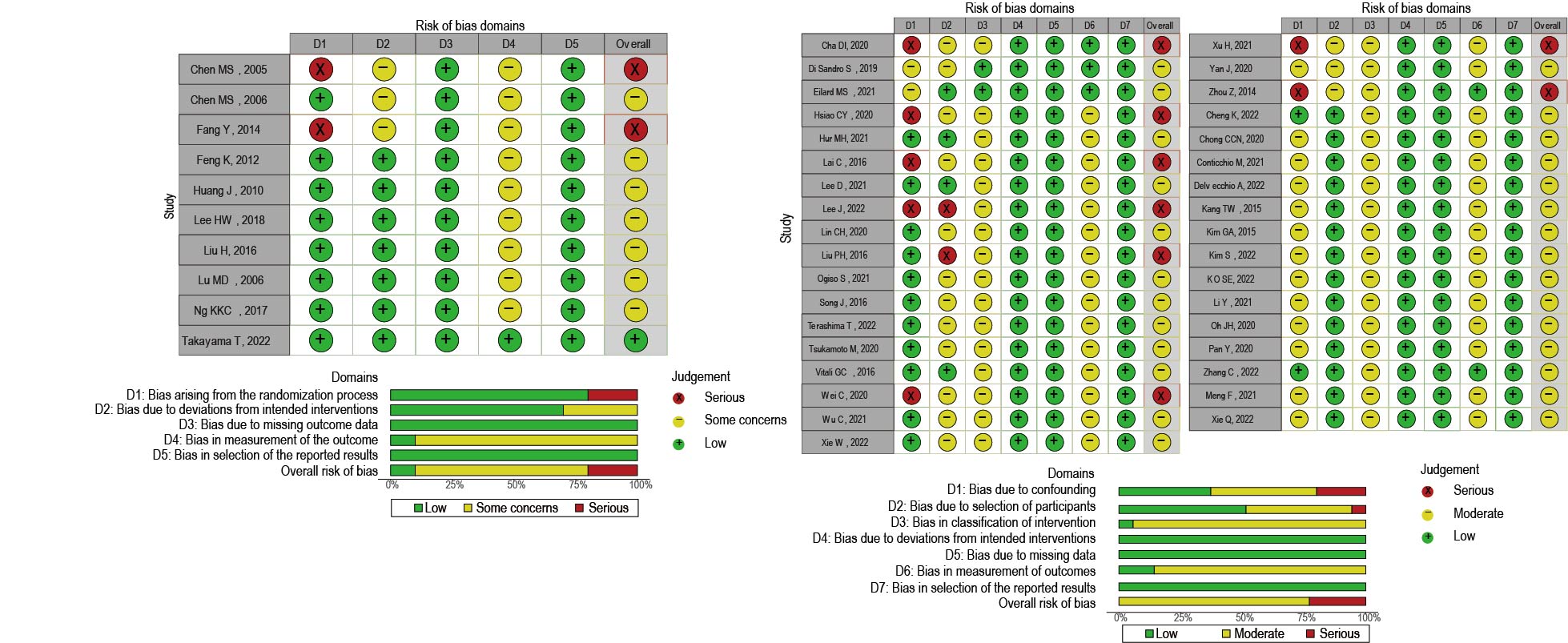

Supplement: Supplementary Figure 1 — Risk of bias evaluation. [file Image1.jpg]

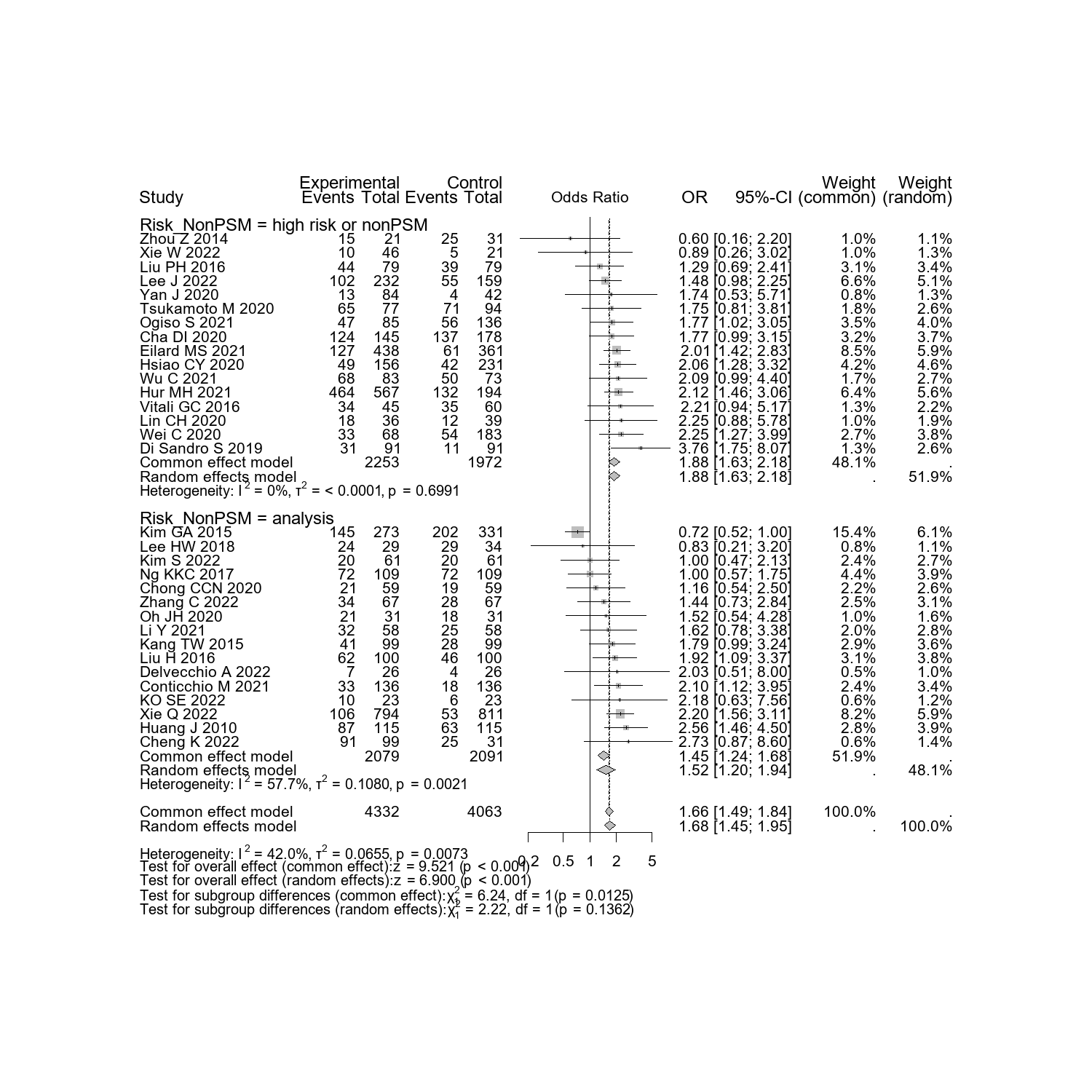

Supplement: Supplementary Figure 2 — Sensitivity analysis of primary outcomes after exclusion of high-risk-of-bias studies and non-propensity-score-matched cohorts. [file Image2.tiff]

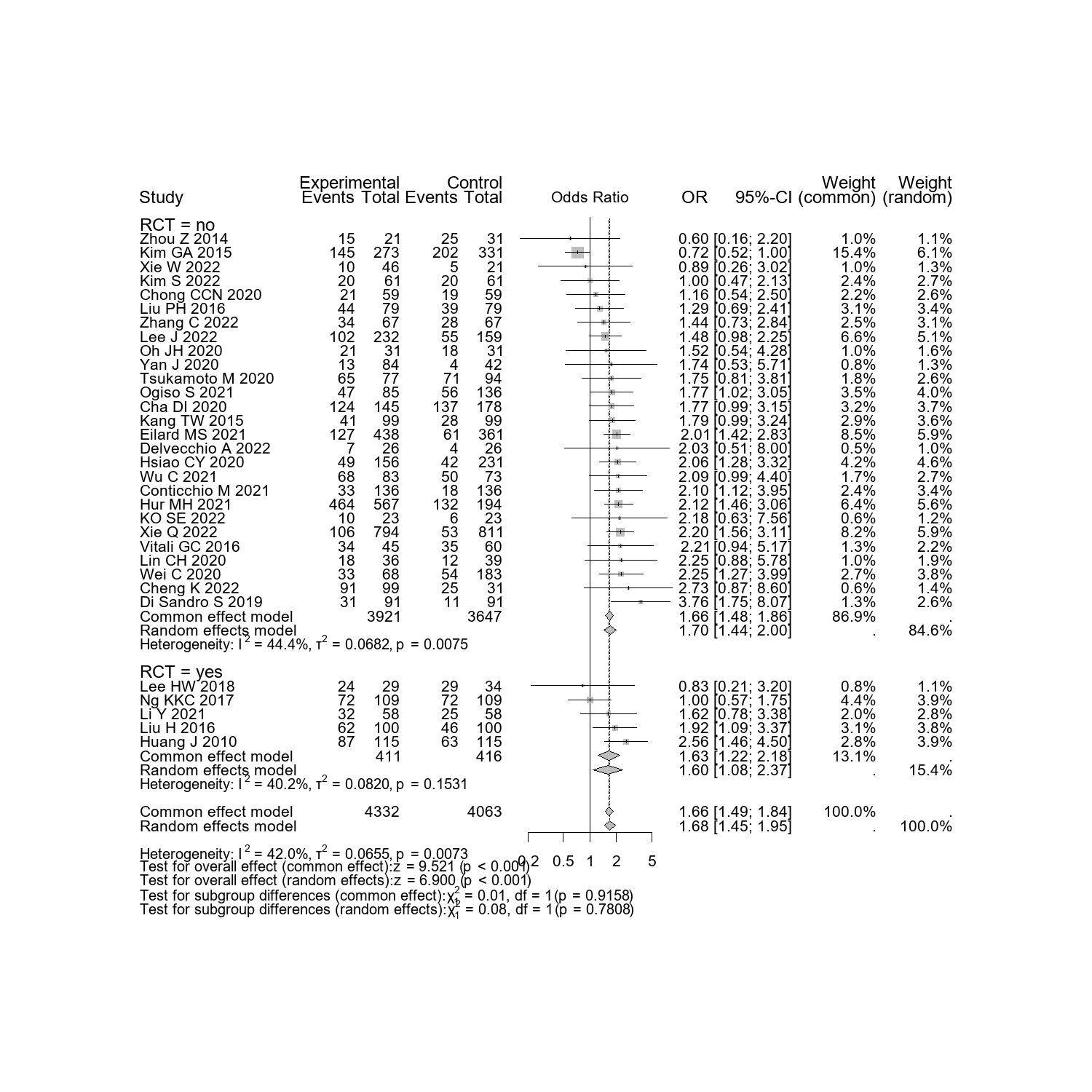

Supplement: Supplementary Figure 3 — Stratified analysis by study design showing maintained advantage of liver resection in both randomized trials and observational studies. [file Image3.tiff]
